# Axillary ultrasound and fine-needle aspiration cytology to predict clinically relevant nodal burden in breast cancer patients

**DOI:** 10.1186/s12957-021-02391-3

**Published:** 2021-09-28

**Authors:** Isabela Panzeri Carlotti Buzatto, Francisco José Cândido dos Reis, Jurandyr Moreira de Andrade, Tamara Cristina Gomes Ferraz Rodrigues, Jéssica Maria Camargo Borba, Amanda Homse Netto, Marina Sconzo Polydoro, Daniel Guimarães Tiezzi

**Affiliations:** Department of Gynecology and Obstetrics, Breast Disease Division, Ribeirão Preto Medical School, University of São Paulo, Avenida Bandeirantes 3.900, Monte Alegre, Ribeirao Preto, São Paulo, SP Brazil

**Keywords:** Breast cancer, Lymph node metastasis, Axillary ultrasound, Fine-needle aspiration cytology

## Abstract

**Background:**

Axillary lymph node involvement is one important prognostic factor in breast cancer, but the way to access this information has been modified over the years. This study evaluated if axillary ultrasound (US) coupled with fine-needle aspiration cytology (FNAC) can accurately predict clinically relevant node metastasis in patients with breast cancer, and thus assist clinical decisions

**Methods:**

This is a cross-sectional study with retrospective data collection of 241 individuals (239 women and 2 men) with unilateral operable breast cancer who were submitted to preoperative axillary assessment by physical exam, US and FNAC if suspicious nodes by imaging. We calculated sensitivity, specificity, and accuracy of the methods. We compared the patient's characteristics using chi-square test, parametrics and non-parametrics statistics according to the variable.

**Results:**

The most sensible method was US (0.59; 95% CI, 0.50–0.69), and the most specific was US coupled with FNAC (0.97; 95% CI, 0.92–0.99). Only 2.7% of the patients with normal axillary US had more than 2 metastatic nodes in the axillary lymph node dissection, against 50% of the patients with suspicious lymph nodes in the US and positive FNAC.

**Conclusions:**

Axillary US coupled with FNAC can sort patients who have a few metastatic nodes at most from those with heavy axillary burden and could be one more tool to initially evaluate patients and define treatment strategies.

## Background

Breast cancer is the most common malignancy among women all over the world and responsible for almost 500,000 deaths each year [1]. Axillary lymph node involvement is one important prognostic factor, but the way to access them has modified over the years [2]. Axillary lymph node dissection (ALND) was performed routinely by William Halsted [3] with great morbidity, especially lymphedema and loss of arm function. In the 1990s, the sentinel node biopsy (SLNB) emerged, leading to the same important prognostic information and minimal morbidity, becoming the standard of care in the management of the axilla in clinically node-negative breast cancer patients [4].

More recently, the role of axillary dissection in sentinel node-positive, early breast cancer patients has been questioned by several trials, including Z0011. They found that ALND did not confer an advantage compared to SLNB in survival nor recurrence, in a subset of T1–2 patients with no palpable adenopathy and 1 or 2 metastatic lymph nodes [5].

Following this trend, non-invasive, less invasive, and costly methods to access the axilla have been studied. Baran et al. found PET/CT and MRI to be highly specific methods (93.18% and 93.75%, respectively), although the sensitivity was not as high (81.03% and 68.57%, respectively) [6]. They also found that combining the methods can enhance specificity to 97.67% and sensitivity to 83.05%. Using diffusion-weighted imaging (DWI) as an advanced technology of MRI can enhance the accuracy of the method in detecting metastatic axillary lymph nodes as demonstrated by Sui et al [7]. It is important to highlight that these methods are expensive and not broadly available.

New technologies such as intradermal microbubbles and contrast-enhanced ultrasound are also currently under investigation [8–10]. It is a promising method to identify the sentinel node and to predict lymph node metastasis before surgery, but larger patient data and multicenter cohort trials are required to establish clinical utility.

Lymph node imaging alone is insufficiently specific to avoid SLNB and proceed directly to ALND; therefore, other strategies had to be developed [11]. The addition of fine-needle aspiration cytology (FNAC) or core needle biopsy to the US enhances the specificity of the method and is being used to assist clinical decisions on breast cancer treatment [12]. It is a relatively low-cost, quick, and well-tolerated procedure associated with minimal morbidity that can evaluate the axillary status pre-operatively, sparing time, and costs of an unnecessary SLNB and helps clinicians to select patients that could benefit from NACT [12–14].

However, the utility of axillary US in recently diagnosed breast cancer patients has been questioned in the past few years because of the practice-changing ACOSOG Z0011 trial [5, 15]. They found that ALND did not confer an advantage compared to SLNB in survival nor recurrence, in a subset of T1–2 patients with no palpable adenopathy and 1 or 2 metastatic lymph nodes [5]. Thus, proceeding to ALND after suspicious US and positive FNAC could lead to overtreatment in this subset of Z0011 patients [15].

The aim of our study was to evaluate if axillary US coupled with FNAC of the suspicious nodes can better predict lymph node metastasis than physical exam in patients with breast cancer. Moreover, we evaluated if axillary US and FNAC can sort patients with a clinically relevant heavy nodal burden from those with minimal or little lymph node involvement.

## Methods

### Study design

**Fig. 1:**
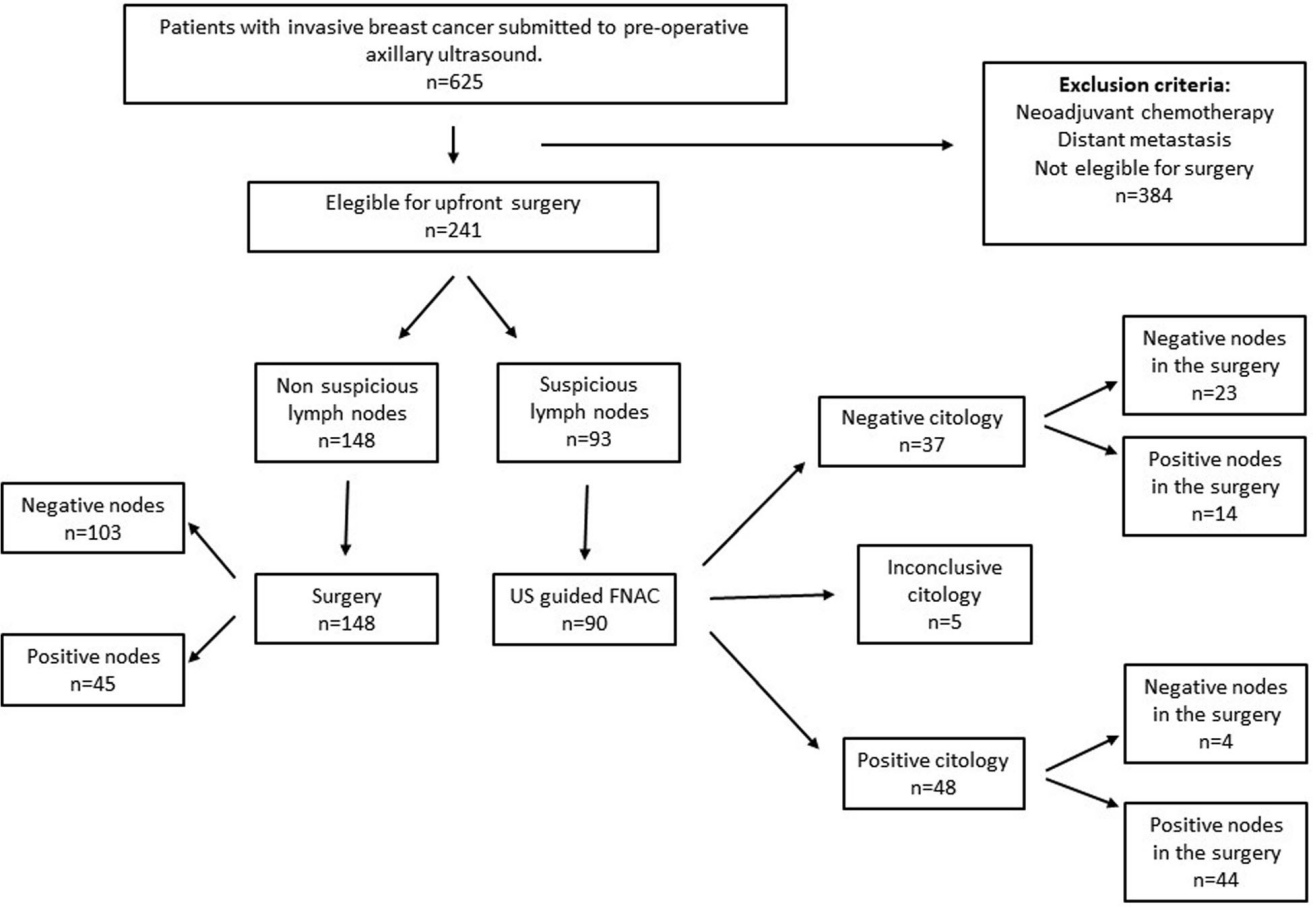
Study design

This is a cross-sectional study with retrospective data collection that included patients with invasive breast cancer who were submitted to preoperative axillary ultrasound. The patients were assessed and treated at the Breast Diseases Division of Hospital das Clínicas of Ribeirão Preto Medical School, University of São Paulo, from August 2015 to November 2019. Patients were excluded if they did not undergo surgery, if they underwent neoadjuvant treatment, if they had distant metastasis, or had tumors that were not primarily from the breast (Fig. 1). Three patients with suspicious lymph nodes in the US that were not submitted to FNAC were included only in the ultrasound analysis. All patients underwent axillary surgery (SLNB or ALND).

### Protocol of preoperative axillary assessment

All patients underwent clinical assessment and axillary ultrasound before treatment. The clinical assessment was performed by an experienced breast surgeon. Clinical axillary status was classified as N0 if no suspicious lymph node was identified, N1 if suspicious movable level I–II axillary lymph nodes were identified, or N2 if palpable suspicious level I–II axillary lymph nodes were clinically fixed or matted [16]. Patients with suspicious sonographic findings underwent FNAC of the most suspicious lymph node. FNAC results were either positive, negative, or inconclusive. For statistical analysis, inconclusive was interpreted as negative (non-positive).

### Ultrasound

**Fig. 2:**
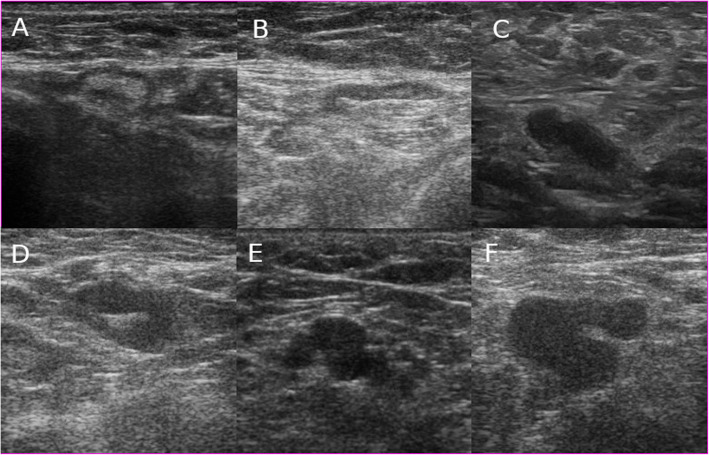
Axillary lymph node ultrasound images. Non-suspicious lymph node in clinically node negative cN0 patient and non-metastatic nodes confirmed after ALND (**A**). Axillary lymph node with eccentric thickening of the cortex in the US, positive FNAC, and one metastatic lymph node in the ALND (**B**). Atypical lymph node in the US in a clinically N1 patient, with positive FNAC and one metastatic node in the ALND (**C**). Atypical lymph node with uniform cortical thickening (> 3mm) in a cN0 patient with positive FNAC and more than three metastatic nodes in the ALND (**D**). Atypical lymph node with compressed fatty hilum and cortical thickening in a cN0 patient with positive FNAC and > 3 metastatic nodes in the ALND (**E**). Irregular node with a mass-like appearance in a cN0 patient, positive FNAC and > 3 metastatic nodes in the ALND (**F**)

The patients were assessed by axillary ultrasound scan using a wide-band linear transducer within at least 4 to 11 MHz range. The ultrasound systems used were Voluson 730 (GE Healthcare, Chicago, USA) until January 2018 and Voluson S10 (GE Healthcare, Chicago, USA) thereafter. All exams were performed by one breast surgeon or one radiologist, both with experience in breast ultrasound. We considered suspicious lymph nodes in the US, those with thickening of the cortex (larger than 3 mm), eccentric medulla, or absent medulla (Fig. 2). The size of the lymph node was not considered because it is less specific than morphology [12, 17–19]. After the ultrasound, the nodes considered suspicious underwent guided FNAC. FNAC was performed in the most representative abnormal node.

### Statistical analysis

We used the R software environment for statistical analysis version 4.0.2 (2020-06-22) and the packages dplyr, furniture, and epiR. Postoperative histology was the gold standard for axillary status. The characteristics of patients with positive and negative nodes were compared using chi-square and non-parametric or parametric statistics according to variable types. For diagnostic performance, we calculated the sensitivity, specificity, and accuracy of the physical exam, axillary US, and axillary US followed by FNAC when US was suspicious for metastasis. To calculate the diagnostic accuracy of ultrasound followed by FNAC, we compared the results of ultrasound and FNAC to the final pathology report (surgical specimen). The preoperative assessment of the axillary lymph nodes was considered negative if the patient had either a negative ultrasound or a suspicious ultrasound and a negative FNAC. The preoperative assessment of the axillary lymph nodes was considered positive if the patient had a suspicious ultrasound with a positive FNAC.

## Results

A total of 241 patients (239 women and 2 men) with histologically confirmed unilateral breast cancer who underwent upfront were included. The characteristics of included patients according to the final axillary status are shown in Table 1.

**Table 1:** Characteristics of patients according to the final axillary status

Variable	Negative nodes	Positive nodes	*p*-value
Number of cases	*n* = 130	*n* = 111	
Age (range)	61.0 (31–88)	58.3 (27–88)	0.081
Tumor size (*T*)			
1a	1 (0.8%)	1 (0.9%)	0.111
1b	19 (14.6%)	5 (4.5%)	
1c	57 (43.8%)	59 (53.2%)	
2	46 (35.4%)	39 (35.1%)	
3	2 (1.5%)	0 (0%)	
4b	4 (3.1%)	3 (2.7%)	
Missing	1 (0.8%)	4 (3.6%)	
Clinical lymph nodes			
N0	110 (84.6%)	65 (58.6%)	< .001
N1	17 (13.1%)	40 (36%)	
N2	1 (0.8%)	6 (5.4%)	
Missing	2 (1.5%)	0 (0%)	
Ultrasound			
Normal	103 (79.2%)	45 (40.5%)	< .001
Suspicious	27 (20.8%)	66 (59.5%)	
Tumor grade			
1	40 (30.8%)	28 (25.2%)	0.601
2	70 (53.8%)	64 (57.7%)	
3	17 (13.1%)	17 (15.3%)	
Missing	3 (2.3%)	2 (1.8%)	
Estrogen receptor			
Negative	23 (17.7%)	12 (10.8%)	0.184
Positive	107 (82.3%)	99 (89.2%)	
Progesterone receptor			
Negative	42 (32.3%)	35 (31.5%)	1
Positive	88 (67.7%)	76 (68.5%)	
HER 2			
Negative	111 (85.4%)	98 (88.3%)	0.637
Positive	19 (14.6%)	13 (11.7%)	
Ki 67			
Negative	41 (31.5%)	39 (35.1%)	0.54
Positive	78 (60%)	60 (54.1%)	
Missing	11 (8.5%)	12 (10.8%)	
Surgery			
Mastectomy	49 (37.7%)	48 (43.2%)	0.457
Tumorectomy	81 (62.3%)	63 (56.8%)	

Forty-six percent of the patients had at least one positive axillary lymph node. The distribution according to age and tumor characteristics (size, grade, and immunohistochemistry biomarkers) were similar between the two groups. The clinical assessment of axillary lymph nodes showed cN1 and cN2 were more frequent among patients with positive axillary lymph nodes.

The individual accuracy of clinical assessment, axillary ultrasound, and ultrasound combined with FNAC to detect axillary lymph node metastasis is shown in Table 2. Adding FNAC to the axillary US did not modify the accuracy, because the increase in the specificity from 79 to 97% was associated with the decrease of sensitivity from 59 to 40%. Since the performance of the FNAC depends on the result of the US, the error of one method interferes with the result of the other, leading to a low sensitivity when combined.

**Table 2:** Accuracy of diagnostic methods for axillary status considering any positive node in the surgery

Method	Clinical	Ultrasound	Ultrasound and FNAC
**Sensitivity**	0.41 (0.32, 0.51)	0.59 (0.50, 0.69)	0.40 (0.30, 0.49)
**Specificity**	0.86 (0.79, 0.91)	0.79 (0.71, 0.86)	0.97 (0.92, 0.99)
**PPVa**	0.72 (0.59, 0.82)	0.71 (0.61, 0.80)	0.92 (0.80, 0.98)
**NPVb**	0.63 (0.55, 0.70)	0.70 (0.62, 0.77)	0.65 (0.58, 0.72)
**Diagnostic accuracy**	0.65 (0.59, 0.71)	0.70 (0.64, 0.76)	0.70 (0.64, 0.76)

*aPPV* positive predictive value, *bNPV* negative predictive value

Twenty-seven out of 91 patients that were clinically node-negative and did not have any suspicious lymph nodes in the US had metastatic nodes in the surgery: 18 patients with one positive node, 2 with micrometastasis, 3 with two positive nodes, and 4 patients with more than two positive nodes.

We identified 99 patients that fit the ACOSOG Z0011 trial criteria: T1 or T2, cN0, submitted to conservative surgery followed by radiation therapy. All of them had a preoperative axillary ultrasound. Only four patients (4%) had suspicious lymph nodes in the US and positive FNAC, but up to two metastatic nodes in the dissection. These four patients were overtreated according to the results from the ACOSOG Z0011 trial, because of the results of the US and FNAC.

On the other hand, we identified 13 patients that were clinically N1 or N2 but had non-suspicious nodes in the ultrasound and underwent sentinel node biopsy. Seven of these patients had no metastatic nodes, 2 had exclusive micrometastatic disease, and 2 had up to two metastatic lymph nodes and did not get axillary dissection. These 11 patients (17% of the *N*+ patients) had treatment deescalated because of the preoperative axillary ultrasound.

**Fig. 3:**
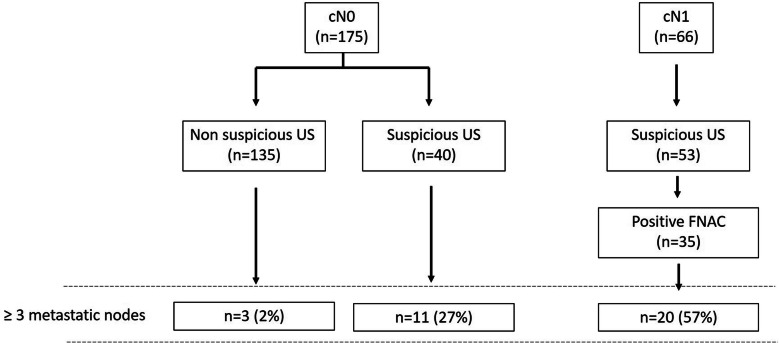
Massive axillary involvement according to the clinical status and US imaging. Percentage of patients with high axillary burden (≥ 3 metastatic nodes in the ALND) according to the clinical assessment, axillary US result, and FNAC result

Suspicious axillary lymph nodes in the US and positive FNAC were associated with a heavier axillary burden. Among the 148 patients with normal axillary ultrasound, only 4 (2.7%) had more than 2 metastatic lymph nodes, while among the 93 with atypical lymph nodes in the US, thirty-two (34%) had more than 2 metastatic nodes (*p* < 0.005). Moreover, among the 48 patients with positive FNAC, 24 (50%) had more than 2 axillary lymph nodes with metastasis (*p* < 0.005). Figure 3 shows the percentage of patients with more than three metastatic nodes in the ALND according to the clinical assessment, axillary US result, and FNAC result.

## Discussion

Axillary lymph node involvement is an important prognostic factor in breast cancer [20–23]. It is well known that the accuracy of physical examination alone is limited [24]; therefore, alternative methods are being investigated to effectively assess the axillary lymph node status with minimal morbidity and low cost. All preoperative methods used in this study (physical exam, axillary US, and FNAC) missed metastatic axillary lymph nodes.

Physical exam and axillary ultrasound are able to predict accurately a low axillary lymph node involvement (less than 3 metastatic nodes), but the false-negative rate of the methods does not obviate the need for SLNB [11, 25]. It is interesting to note that almost 96% of our patients with no suspicious nodes in the physical exam and the US had less than three positive nodes in the surgery. Now, it is not yet clear if that is enough information to tailor the adjuvant treatment [26]. The ongoing SOUND trial (NCT02167490) is prospectively evaluating if preoperative imaging of the axilla in early breast cancer patients can identify patients with clinically relevant nodal burden and safely spare patients from SLN biopsy [27].

On the other side, patients with palpable adenopathy are at a higher risk of having massive axillary involvement and are managed either with upfront ALND or, increasingly, neoadjuvant chemotherapy [28]. Our data are in accordance since half of our cN1/N2 patients had positive nodes at the final pathology assessment even with a normal ultrasound imaging. It is relevant to highlight the small number of patients in this scenario, since most of the cN1/N2 patients in our institution go to neoadjuvant therapy.

The sensitivity of the US alone is admittedly low, varying from 26.4 to 75.9%, as well as the sensitivity of other imaging methods such as PET-CT that varies from 35 to 85% [11]. Our findings are in accordance with the literature; the sensitivity of the US alone to detect axillary lymph node metastasis was 59%.

Before deciding about scalonating treatment (local or systemic) because of a suspicious lymph node image, it is essential to confirm the finding with FNAC or core needle biopsy (CNB) [29]. The PPV of the US alone is not high enough to directly proceed to ALND without tissue sampling, ranging from 56 to 90% [29, 30]. Our data are in accordance; we found a PPV of US of 71%. FNAC was particularly useful though to confirm a metastatic lymph node when ultrasound was suspicious since the specificity of US was 79% while the specificity of US coupled with FNAC was 97%.

The absence of neoplastic cells on FNAC in ultrasound suspicious nodes did not exclude completely a malignant node. In our study, we had 17/61 patients with metastatic nodes in the surgical specimen that had suspicious nodes in the US and a false-negative FNAC result, leading to a false-negative (FN) rate of 28%. This is a recognized limitation of the method due to inadequate sampling, preparation, or interpretation of the material. In the literature, the FN rate of US-FNAC ranges from 9 to 41% [31]. In daily practice, US-FNAC/CNB are performed only for patients with suspicious nodes in the US, which leads to an even higher FN rate if the images are not interpreted properly [31]. Despite the great effort made to diminish the false-negative rate of FNAC, it remains too high to omit definitive surgical staging of the axilla [25]. A meta-analysis comparing the diagnostic accuracy of FNAC and CNB showed an increased sensitivity for the latter and similar very high specificity for both methods [31]. FNAC has various advantages though, including minimal invasiveness, safety, and low cost [31].

Patients with suspicious US and positive FNAC were more likely to have a higher disease burden than those with positive sentinel nodes alone and therefore represent a distinct patient population that should be addressed carefully [32].

The accuracy of the method is high enough to sort patients that have a few metastatic lymph nodes at most from those with a heavy axillary burden (three or more positive nodes) [15, 20, 26]. Our findings are highly in accordance, since among the clinically node-negative patients with normal US, only 2% had more than 3 metastatic lymph nodes in the surgical specimen while among the patients with suspicious nodes in the ultrasound and positive FNAC, 57% had massive axillary lymph node involvement. In this context, the methods could help sort patients with a high axillary burden that can benefit from neoadjuvant therapy, including deescalating axillary surgery.

To our understanding, axillary evaluation of breast cancer patients is an evolving topic, and axillary US coupled with FNAC could be a less invasive, highly available strategy used to define therapy.

It is relevant to highlight the possible limitations of the methods, including a reasonably high false-negative rate even when combining physical exam and US [12] and the possible axillary surgical overtreatment created by a positive FNAC in the ACOSOG Z0011 context [15]. The extent of this harm is not consensual yet. While some authors found a great number of patients undergoing unnecessary ALND because of a suspicious image and positive cytology/pathology [33], others observed that patients with a high nodal burden are those that have positive FNAC or CNB [34]. Our findings are in accordance with the latter. Only 4% of the patients with suspicious US and positive FNAC would have fit the ACOSOG Z0011 criteria and were overtreated with ALND in our study.

This study has some limitations that need to be addressed, such as the retrospective nature. Another relevant limitation of the present study is that ultrasound is highly operator dependent [35], and even though it is interesting to know the results of the methods in the daily practice, the reproducibility was not evaluated. Lastly, the lymph nodes submitted to US-guided FNAC were not routinely clipped, and therefore, it is not possible to conclude that they were retrieved in the axillary surgery.

It would be interesting to look forward to whether the association of axillary US to other imaging techniques such as mammography, MRI, and PET-CT could enhance the sensibility of the method and assist clinical decisions.

## Conclusion

Axillary US coupled with FNAC can sort patients that have a few metastatic nodes at most from those with heavy nodal burden and could be one more tool to initially evaluate patients and define treatment strategies as well as sort patients that can benefit from neoadjuvant therapy.

## Data Availability

The datasets used and/or analyzed during the current study are available from the corresponding author on reasonable request.

## Abbreviations

ALND: Axillary lymph node dissection
CNB: Core needle biopsy
cN0: Clinically node-negative
DWI: diffusion-weighted imaging
FN: False negative
FNAC: Fine-needle aspiration cytology
MRI: Magnetic resonance imaging
NACT: Neoadjuvant chemotherapy
NPV: Negative predictive value
PET/CT: Positron emission tomography/computed tomography
PPV: Positive predictive value
SLN: Sentinel lymph node
SLNB: Sentinel lymph node biopsy
US: Ultrasound
